# Contrasting risk patterns from human hunters and a large carnivore influence the habitat selection of shared prey

**DOI:** 10.1007/s00442-025-05742-z

**Published:** 2025-07-01

**Authors:** G. Ausilio, C. Wikenros, H. Sand, O. Devineau, P. Wabakken, A. Eriksen, M. Aronsson, J. Persson, K. M. Mathisen, B. Zimmermann

**Affiliations:** 1 Faculty of Applied Ecology, Agricultural Sciences and Biotechnology, University of Inland Norway, Campus Evenstad, 2480 Koppang, Norway; 2 Grimsö Wildlife Research Station, Department of Ecology, Swedish University of Agricultural Sciences, 73993 Riddarhyttan, Sweden

**Keywords:** *Alces alces*, Human-dominated landscape, Anti-predator behaviour, *Canis lupus*, Harvest, Predation

## Abstract

Spatial patterns of human hunting and predation risk are mediated by the physical landscape, with hunting risk often associated with different habitat features than those linked to large carnivores. Risk from hunters and carnivores also varies over time, and prey may adjust anti-predator strategies accordingly. We used GPS data from 17 female moose (*Alces alces*) tracked over two fall–winter seasons (2018–2020) in south-central Scandinavia to study moose habitat selection in response to diel and seasonal variation in risk from wolves (*Canis lupus*) and human hunters. Predation risk was quantified using spatial models based on known wolf and hunter kill-sites. We applied resource selection functions and Generalized Additive Mixed Models to model moose habitat selection in response to wolf and hunting risk. Moose avoided high-risk hunting areas during the day throughout the hunting season but relaxed this avoidance at night and after the season ended. In contrast, we found no evidence that moose adjusted habitat use in response to diel or seasonal variation in wolf predation risk. These results suggest that human hunting was the primary driver of moose habitat selection during the hunting season, becoming less relevant when hunting ceased. Our findings highlight the dominant role of human risk in shaping prey behaviour and the importance of accounting for hunting when evaluating predator–prey dynamics in human-dominated systems. An increased understanding of the risk effects arising from humans and large carnivores on prey can deepen our understanding of the ecological roles of predators and humans.

**Supplementary Information:**

The online version contains supplementary material available at 10.1007/s00442-025-05742-z.

## Introduction

Predation risk can induce changes in prey behavior and physiology (Lima 1998; Laundré et al. 2001; Creel and Christianson 2008), leading to various anti-predator responses, including shifts in habitat selection (Fortin et al. 2005), activity patterns (Hudgens and Garcelon 2011; Tambling et al. 2015), and movement behavior (Laundré et al. 2001; Sih and McCarthy 2002). These defensive strategies, however, often come at a cost—increasing physiological stress and reducing foraging efficiency (Morgantini and Hudson 1985)—which can negatively affect growth (Pangle et al. 2007), reproduction (Boonstra et al. 1998; Cherry et al. 2016), and survival (Lima and Dill 1990; Gehr et al. 2018). To balance these trade-offs, prey must continually adjust their behaviour based on current predation risk (Lima and Dill 1990). Spatiotemporal variation in predation risk is shaped by a predator’s space use, hunting mode, and interactions with habitat features, photoperiods, and environmental conditions, all of which influence predation efficiency (Schmitz 2005; Preisser et al. 2007; Atwood et al. 2007; Miller et al. 2014; Gaynor et al. 2021). Differences in predator diel activity (Monterroso et al. 2014; Gaynor et al. 2021) can create temporal refuges that prey exploit during safer periods (Palmer et al. 2022). For instance, elk (*Cervus elaphus*) have been observed selecting riskier habitats when wolves (*Canis lupus*) are less active (Kohl et al. 2019).

Conversely, predators may adjust their own activity or habitat use to match prey behaviour and improve hunting success (Harrington and Mech, 1982; Fuller, 1991; Theuerkauf et al., 2003). This may be especially advantageous in systems with low prey-to-predator ratios, where prey is harder to locate and requires more targeted effort (Stephen and Krebs, 1986). In contrast, predators operating in areas with high prey-to-predator ratios may not face similar challenges in finding prey and may therefore have less need to modify their activity to match that of their prey (Eriksen et al., 2011).

Humans have become “super predators” in many ecosystems (Darimont et al. 2015; Smith et al. 2017), with game harvesting now being a major cause of mortality for many ungulates (Allendorf et al. 2008). As a result, hunting strongly influences prey behavior and distribution (Proffitt et al. 2009), often reducing fitness (Grignolio et al. 2007; Neumann et al. 2009) and driving the development of anti-predator strategies (Lima and Dill 1990; Lima 1998; Caro 2005; Creel and Christianson 2008). While risk effects of both large carnivores and human hunters have been studied, most research has focused on ambush predators like lynx (*Lynx lynx*) and mountain lions (*Puma concolor*) (Lone et al. 2014; Norum et al. 2015; Gehr et al. 2018; Gaynor et al. 2021). Cursorial predators like wolves, offer less predictable cues, often resulting in weaker habitat-mediated fear responses (Kauffman et al. 2010; Thaker et al. 2011; Schmidt and Kuijper 2015). However, how prey navigate and balance the risks posed by both human hunters and cursorial predators remains poorly understood (Theuerkauf and Rouys, 2008; Proffitt et al. 2009).

In south-central Scandinavia, moose (*Alces alces*) are exposed to predation from wolves (Sand et al. 2005, 2008), brown bears (*Ursus arctos*) (Swenson et al., 2007), and human harvest (Lavsund et al. 2003; Wikenros et al. 2015a). Wolves were absent for over 150 years before recolonizing the region in the 1980 s (Wabakken et al. 2001), during which time human hunting replaced natural predation (Sand et al. 2006). Despite their return, evidence for risk effects from wolves remains limited (Nicholson et al., 2014; Månsson et al., 2017; Sand et al. 2021), likely because human harvest remains the dominant mortality source for moose (Sand et al. 2006).

Wolves and hunters impose contrasting spatiotemporal risk patterns: hunting occurs during the day and only in autumn, while wolf predation is primarily nocturnal and year-round (Ausilio et al. 2022). Using kill location data, Ausilio et al. (2022) created spatial risk maps showing that hunting risk was highest in clear-cuts/young forests, bogs, near roads, in flat terrain, and areas with low building density. In contrast, wolf risk was associated with rugged terrain, clear-cuts, and areas farther from bogs. However, it remains unclear how moose adjust habitat selection in response to these varying spatiotemporal risk patterns. Given that hunting risk is highly predictable and concentrated in time, it likely drives daytime behavior during the hunting season (Kuijper et al. 2016), while wolf risk may influence nocturnal behavior or behavior outside the hunting season (Sönnichsen et al. 2013; Lone et al. 2014; Kuijper et al. 2016).

In this study, we use the risk maps from Ausilio et al. (2022) as a predictor to examine whether moose adjust habitat selection in response to temporal variation in human hunting and wolf predation risk. We expected seasonal and diel habitat selection to align with each predator’s activity patterns (e.g., hunting season vs. non-hunting season, day vs. night). We expected habitat use to reflect each predator’s activity patterns—specifically, that moose would (P1) avoid high hunting risk areas during the day in the hunting season and (P2) avoid high wolf risk areas at night year-round.

## Materials and methods

### Study area

**Fig. 1:**
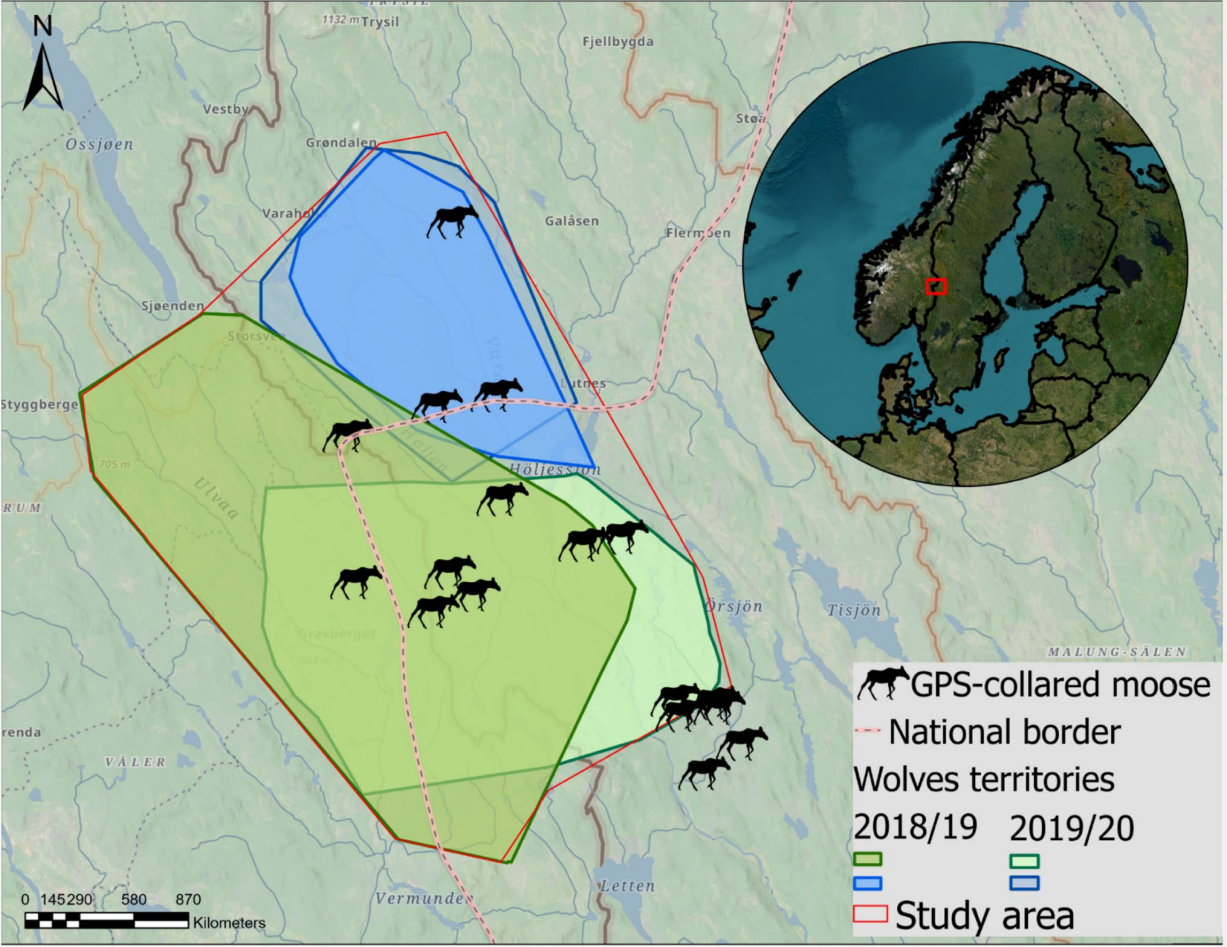
Study area in south-central Scandinavia, spanning the Swedish-Norwegian border (dotted pink line), where data were collected from mid-August to April over two consecutive fall-winter seasons (2018–2019 and 2019–2020). The map shows the spatial overlap between two wolf territories (2018/19 and 2019/2020) and the location of 17 GPS-collared female moose (black silhouettes). The inset in the top right corner provides a zoomed-out satellite view of northern Europe, with a red square indicating the study region

The study was conducted from mid-August to April over two consecutive fall-winters (2018/19 and 2019/20) along the Swedish-Norwegian border (60°33’–61°15’N, 11°45’–12°55’E), covering 1699 km^2^ in Norway and 969 km^2^ in Sweden (Fig. 1). The landscape consists mainly of boreal forest dominated by Scots pine (*Pinus sylvestris*), Norway spruce (*Picea abies*), and birch (*Betula* spp.) (Antonson 2011; Christiansen 2014). Elevation ranges from 125 to 743 m, creating a north–south climatic gradient, with snow-rich areas (30–95 cm) in the north and snow-poor areas (0–35 cm) in the south (Zimmermann et al., 2022). The region is highly accessible due to an extensive network of gravel roads (mean road density: 0.84 km/km^2^), except for some unploughed roads in winter.

Wolves have been present in the study area since the 1980 s (Wabakken et al. 2001). During the study period, two wolf territories were documented within the area: the first established in 2014/2015 and the second in 2015/2016 (Fig. 1). Pack sizes varied between 3–4 wolves (2018/19) and a territorial pair (2019/20) in one territory and a territorial pair (2018/19) and 5–7 wolves (2019/20) in the other (Svensson et al. 2019; Wabakken et al. 2020) (Fig. 1). The Scandinavian wolf population, which was extinct in the 1960 s, was re-established in the 1980 s from the Finnish-Russian population (Wabakken et al. 2001). By 2018/19 and 2019/20, the population reached 380 (95% CI = 300–494) and 450 (95% CI = 356–585) individuals, respectively (Svensson et al. 2019; Wabakken et al. 2020). No legal culling occurred in the study area during the study period. Brown bear density in the study area was low, with an average of 0.2 bears/1000 ha (Bischof et al., 2020).

The average winter moose density was 1.25–1.27 moose/km^2^, estimated via faecal pellet counts (Zimmermann et al. 2019). Hunting teams within management units are required to report their annual harvests (Wikenros et al. 2020). The hunting season runs from late September to December in Norway and from early September to February in Sweden. During the 2018/19 and 2019/20 hunting seasons, 409 and 472 moose were harvested, respectively (Ausilio et al. 2022).

### Data collection

During the study period, 18 female moose were immobilized by helicopter darting (Arnemo and Evans 2017) and fitted with GPS collars (Vectronics Survey and VertexPlus, Vectronic Aerospace GmbH). Handling protocols complied with ethical guidelines for wildlife research in Sweden (C281/6, C315/6) and Norway (ID 15170). Collars were programmed to record one GPS position every two hours, including daylight status at each fix. Daylight is determined automatically by the GPS collars, which use a built-in function based on local sunrise and sunset times. GPS data were screened for errors using the non-movement method (Bjørneraas et al. 2010), applying a speed limit of 1.5 km/hr, distance parameter (Δ) of 100 km, and turning angle (θ = − 0.97). After quality control, data from 17 female moose were retained, totalling 63,551 GPS positions collected from September to April over two years.

### Hunting risk and wolf predation risk

**Fig. 2:**
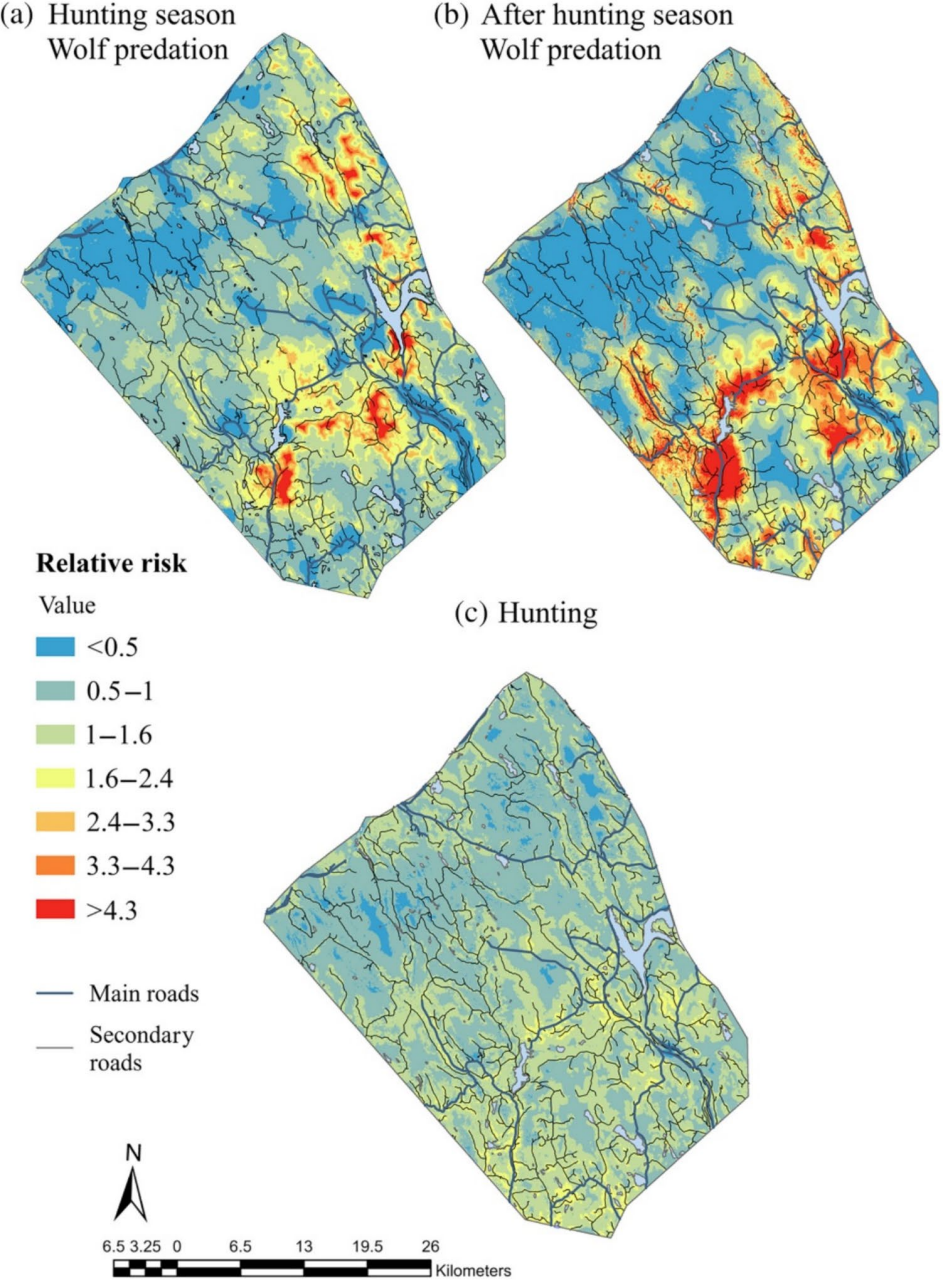
Predicted relative risk maps from Ausilio et al. (2022) from the same study area, illustrating cross-border spatial variation in wolf predation and moose hunting risk. Panel **a** shows the relative risk of wolf predation during the hunting season, panel **b** after the hunting season, and panel **c** the relative risk of moose harvest by hunters. Risk values are expressed relative to the average: a value of 5 indicates a fivefold higher-than-average risk at that location, while a value of 0.5 indicates half the average risk. These maps were produced using coefficients from the top-ranked models of that study, based on wolf- and hunter-killed moose during and after the hunting season. We used these risk maps to extract relative risk values of wolf predation and hunting for each GPS-collared moose included in our analysis

To assess how moose respond to spatial risk patterns, we incorporated hunting and wolf predation risk maps developed specifically for this study area (Ausilio et al. 2022; Fig. 2). These maps were created using logistic regression models based on hunter-killed and wolf-killed moose sites, with risk predicted as a function of environmental and anthropogenic factors known to influence both predation and hunting pressure. Key variables included distance to bogs, clear-cuts/young forests, main and secondary roads, building density, and terrain ruggedness, all of which are linked to predator hunting efficiency and human accessibility. The resulting risk maps were generated at a 25 × 25 m resolution, reflecting the fine-scale environmental heterogeneity that influences both moose movement and predation/hunting risk. For example, clear-cuts and roads—key determinants of hunting risk—vary at a similar spatial scale, and terrain ruggedness, which affects wolf predation efficiency, also changes over short distances. The maps depict relative risk values, where a score of 5 indicates a fivefold higher likelihood of moose mortality in a given location compared to the average risk (score 1) (Fig. 2). Results from Ausilio et al. (2022) showed that hunting risk was highest near bogs, roads, and clear-cuts, while wolf predation risk was associated with clear-cuts, young forests, and rugged terrain. By integrating these risk maps, we were able to test how moose adjust their habitat selection based on both spatial and temporal variations in predation risk.

### Habitat selection analysis

We estimated seasonal home ranges using 95% minimum convex polygons (MCPs) with the amt package in R (Signer et al. 2019), defining two periods: during the hunting season (Sept 1 – Jan 15) and after the hunting season (Jan 16 – Apr 30). The average home range size was 35.5 km^2^ during and 23.5 km^2^ after the hunting season (see Fig. 1, Supplementary Material). To investigate moose habitat selection, we applied resource selection functions (RSFs) (Manly et al. 2002; Morris et al. 2016), a widely used approach that compares spatial attributes of used locations to those of randomly selected available locations within an animal’s home range (Manly et al. 2002). Within each home range, we generated randomly available locations equal in number to actual moose locations (1:1 ratio of used to available locations per moose, season, and time of day). We then extracted the corresponding relative risk of hunting and wolf predation for each GPS and random location.

For analysis, we coded GPS locations as 1 (used) and random locations as 0 (available) and modelled habitat selection using generalized linear mixed models (GLMMs) with a binomial distribution (logit link) in the lme4 package (Bates et al., 2015). Since multicollinearity between hunting and wolf predation risk was low (Pearson’s r < 0.5), both risks were included in the same model. We examined seasonal and diel habitat selection by modelling the binary response variable as a function of hunting risk, wolf predation risk, diel period (day/night), all two-way interactions, and the three-way interaction between diel period, wolf risk, and hunting risk. We generated separate models for each season (during and after hunting) due to convergence issues when including season as a covariate. Individual moose nested within the year was included as a random factor, and continuous covariates were standardized by subtracting the mean and dividing by the standard deviation (Gelman and Hill, 2007).

To validate the models, we conducted K-fold cross-validation for RSFs (Boyce et al., 2003) using the cvms package (Olsen et al., 2025). We partitioned the dataset into four folds (k = 4), ensuring ordered subsets, and applied *cross_validate*, which provided multiple model performance metrics.

### Seasonal change in habitat use

To examine non-linear patterns of habitat use over the study period (September–April), we modelled the relative hunting and wolf predation risk of used locations over time using Generalized Additive Mixed Models (GAMMs). This approach allowed us to capture temporal trends in absolute risk values selected by moose while providing greater flexibility than linear models for analysing complex relationships between predictors and responses (Hastie and Tibshirani 1990).

We extracted relative hunting and wolf predation risk from the risk maps of Ausilio et al. (2022) and modelled the relative risk of used locations over time. Time was calculated as the number of days from August 15, providing a two-week margin to account for potential shifts in prey responses. We modelled hunting risk and wolf risk separately, including time, diel period (day/night), and all two-way interactions as fixed factors.

## Results

### Habitat selection

**Fig. 3:**
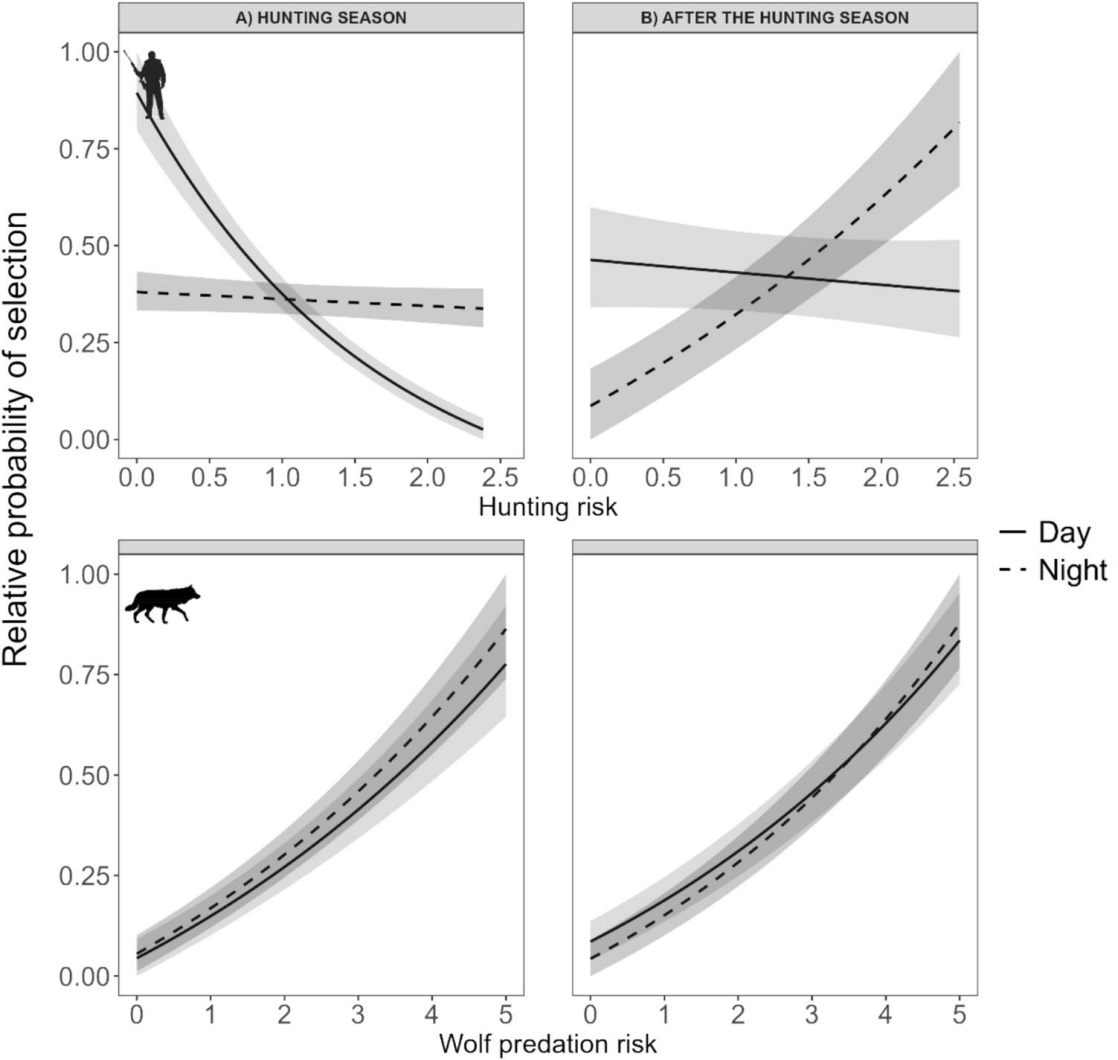
Relative probability of selection by moose (N = 17) in relation to hunting risk and wolf predation risk for day and night (expressed as odds ratio values) during the hunting season (September 01 – January 15; panel a); and after the hunting season (January 16 – April 01; panel b), in south-central Scandinavia (2018/19 and 2019/20). The relative probability was estimated using generalized linear mixed regression, where moose GPS locations were compared to random locations within each moose home range during day and night (but plotted together for visual purposes). Hunting risk and wolf predation risk were estimated using hunter- and wolf-killed moose compared to random locations (ratio 1:1) (see Ausilio et al. 2022 for more information)

Moose exhibited diel and seasonal differences in habitat selection in response to hunting and wolf predation risk. As predicted, during the hunting season, moose selected habitats with lower hunting risk during the day compared to night (P1; Table 1, Fig. 3A). However, contrary to our prediction (P2), moose selected areas of higher wolf predation risk at night, both during and after the hunting season (Table 1, Fig. 3).

**Table 1:** Logistic mixed-model regressions investigating the effect of human hunting and wolf predation risk on moose

Hunting season			
Variable	β estimate	Standard error	p-value
*Wolf risk*	− 0.190	0.043	< 0.001
*Hunting risk*	− 1.149	0.082	< 0.001
Wolf risk*Night	0.105	0.055	0.056
Hunting risk*Night	0.670	0.100	< 0.001
Hunting risk*Wolf risk	0.338	0.044	< 0.001
Hunting risk*Wolf risk*Night	− 0.075	0.055	0.174
*Night*	− 0.736	0.104	< 0.001

After the hunting season			
	β estimate	Standard error	p-value
*Wolf risk*	0.047	0.026	0.075
*Hunting risk*	− 0.312	0.072	< 0.001
Wolf risk*Night	0.103	0.036	0.004
Hunting risk*Night	0.381	0.099	< 0.001
Hunting risk*Wolf risk	0.109	0.020	< 0.001
Hunting risk*Wolf risk*Night	− 0.060	0.028	0.033
*Night*	− 0.557	0.113	< 0.001

(N = 17) habitat selection (moose GPS locations versus random locations) during (September 01 – January 15) and after the hunting season (January 16 – April 30) in south-central Scandinavia. Estimates (b), standard error (SE) and p-values are provided for each model

After the hunting season, moose no longer adjusted habitat selection based on hunting risk during the day, but their selection for riskier hunting areas increased at night (Table 1, Fig. 3B). The two-way interaction between hunting and wolf predation risk was significant in both seasons. The probability of moose selecting habitats with high wolf predation risk increased with increasing hunting risk, irrespective of time of day (the three-way interaction between diel period and both risks was not significant). Both models, during and after the hunting season, showed moderate performance in K-fold validation, with balanced accuracy scores of 0.55 and 0.57, respectively.

### Habitat use

**Fig. 4:**
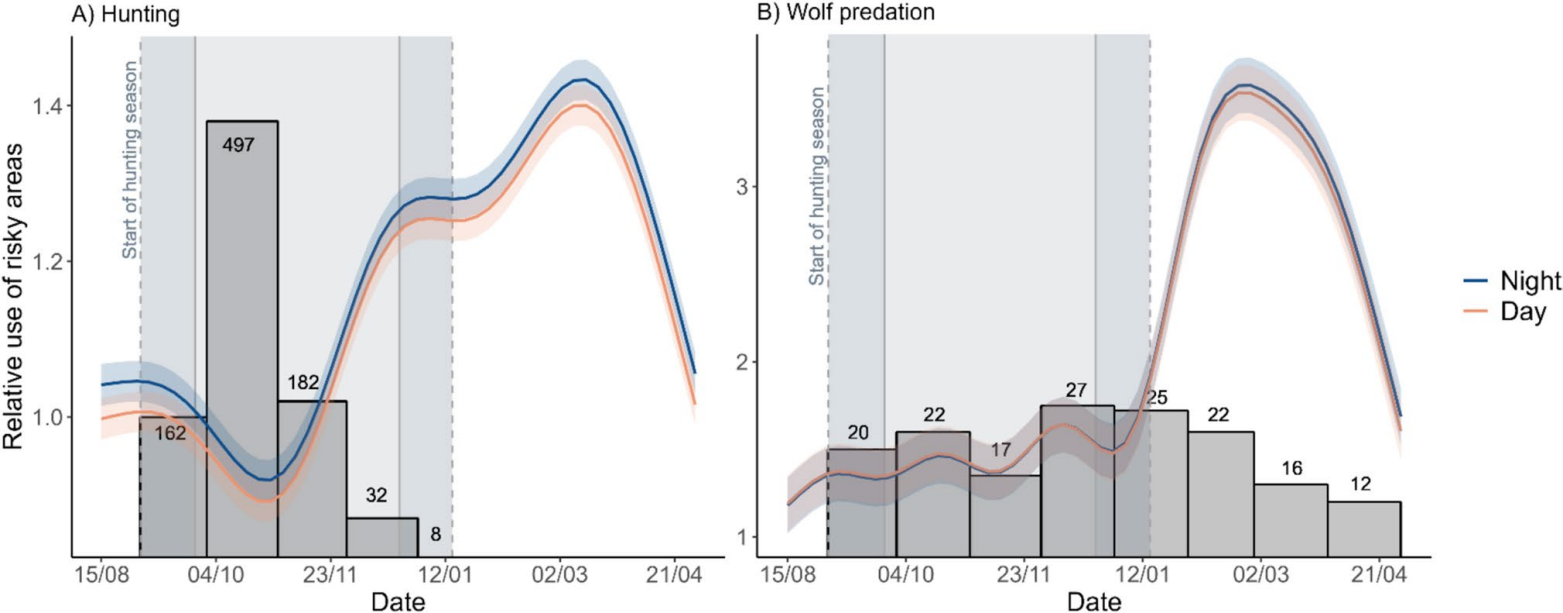
Relative hunting risk (± SE) and wolf predation risk (± SE) of used locations during day and night for moose (N = 17) in south-central Scandinavia throughout half a year (1st September until 30th April) in relation to time during the year. A two-week margin was added to the analysis to account for changes in prey responses prior to the start of the hunting season (the x-axis starts on the 15th of August). The darker and lighter blue shaded areas indicate the beginning of the hunting season in Sweden (1st of September) and Norway (25th of September), respectively. The grey-shaded bars represent the total number of moose shot or killed by wolves per month within our study area during two hunting seasons (numbers reported in the bars)

The nonlinear GAMMs used to describe moose use of risky areas showed that the lowest exposure to hunting risk was during mid-October, which coincides with the most intense hunting season (Fig. 4A). As the hunting season progressed, moose increasingly used areas of higher hunting risk, only to decrease in late April again (Fig. 4A). Time of day was significant only as a linear term, with moose selecting areas of lower hunting risk during the day compared to the night (Table 1 in Supplementary Material). The exposure to wolf predation risk was lowest during the hunting season and highest during late February and early March (Sep – January; Fig. 4B).

## Discussion

Our study shows that moose habitat selection is primarily shaped by hunting risk, which is the most predictable risk. During the hunting season, moose consistently avoided high-risk areas during the day, when hunting occurs, but did not avoid these areas at night when hunting is not allowed, nor possible. This behaviour was most pronounced early in the season, when most moose were harvested, and weakened as the season progressed. With increasing hunting risk, moose selected areas with higher wolf predation risk, aligning with contrasting temporal risk patterns between hunters and wolves (Ausilio et al. 2022). After the hunting season, moose used areas of higher hunting risk at night, but selection during the day remained unaffected. Overall, moose selected areas with higher wolf predation risk, suggesting they adjust their anti-predator behaviour primarily in response to hunting risk (Kuijper et al. 2016; Lone et al. 2014), rather than to wolf predation risk.

While these findings provide valuable insights, we acknowledge the modest predictive power of our models. This may be due to unmeasured variables affecting habitat selection patterns in moose like food availability. As noted in previous studies, habitat selection models often have poor predictive abilities (Torres et al., 2015; Fieberg et al. 2021). Despite this, our results align with and contribute to a growing body of literature showing that moose in Scandinavia have not exhibited expected anti-predator behavior towards wolves since their recolonization 30–40 years ago (Sand et al. 2006; Nicholson et al., 2014; Sand et al. 2021, Eriksen et al 2008; 201, Wikenros et al. 2009; 2016, Månsson et al. 2017).

A plausible explanation for the observed pattern is that in our study system, individual moose face relatively low wolf predation risk because wolf predation is minimal compared to other sources of moose mortality (Sand et al. 2021, 2025). During our study period, most kill sites were hunter-kill sites (85%), while wolf predation accounted for roughly 15% of the kill sites (Ausilio et al. 2022). The average predator-to-prey ratio in our study system is low (1:630, based on a moose density of 1.3/10 km^2^ in winter and an average wolf territory size of 1000 km^2^; Mattisson et al. 2013), meaning moose face relatively low predation pressure from wolves (Sand et al. 2012, 2025). The low wolf-to-moose ratio results in fewer than 10% of moose being killed by wolves annually on average (Zimmermann et al. 2015; Sand et al. 2025), and a low frequency of encounters between wolves and individual moose (Eriksen et al. 2008, Wikenros et al. 2016). Consequently, wolves exert weak selection pressure on moose habitat selection, supporting our finding that moose did not consistently avoid areas of high wolf predation risk. In contrast, human hunting poses a much higher mortality risk (~ 2–2.5 times greater than wolf predation; Sand et al. 2025), making it the dominant driver of moose habitat selection. This, in combination with the fact that hunting in Scandinavia has functionally replaced natural predation by wolves on moose during the last century (Sand et al. 2006), is likely the ultimate cause of the moose selection pattern found in this study.

Gasaway et al. (1992) found that in areas with high moose-to-wolf ratios, moose populations were more influenced by food availability than by wolf predation. Similarly, Vucetich et al. (2011) demonstrated that in predator–prey systems with low predator-to-prey ratios, prey population growth is more strongly influenced by resource availability than by predation pressure. In these systems, predation is often compensatory, with prey populations regulated more by resource competition and density-dependent factors than by direct predation (Bowyer et al., 2005, 2013; Person et al. 2001). If predation pressure is low due to infrequent predator–prey encounters, prey may prioritize foraging over predator avoidance. Sand et al. (2021) reported that Scandinavian moose continue using high-risk habitats despite wolf presence, likely due to the low overall probability of predation. Similarly, Lone et al. (2017) found that roe deer (*Capreolus capreolus*) continued using lynx-risky habitats during winter, suggesting that food scarcity can override anti-predator behaviors. In low predator-to-prey ratio systems, the trade-off between foraging and avoiding predators becomes less pronounced, as the immediate predation risk is reduced (Bowyer et al., 2005). Our findings support the notion that prey respond most strongly to the most predictable risk, which in human-dominated landscapes is often hunting (Kuijper et al. 2016). Therefore, the risk effects imposed by hunters are expected to be the main determinant of prey responses during the hunting season, while decreasing in importance when hunting is not permitted.

This study included only adult female moose, which constrains the generalizability of our findings to the broader moose population. Because male moose are often more heavily targeted by hunters than by wolves, they may exhibit even stronger spatial avoidance of hunting risk. However, given that wolves primarily prey on calves and subadults (Sand et al. 2005; 2008), the exclusion of males is unlikely to alter our overall conclusions regarding weak responses to wolf predation risk. Nonetheless, future studies incorporating both sexes could offer valuable insight into potential sex-specific differences in risk perception and behavioural trade-offs, particularly in systems where hunting pressure is sex-biased.

This study adds to the growing body of literature documenting the contrasting effects of human hunters and large carnivores on prey (Proffitt et al. 2009; Lone et al. 2014; Norum et al. 2015; Gaynor et al.^2021^). With the return of large carnivores to Europe and North America (Wabakken et al. 2001; Chapron et al., 2014), wild ungulates are now exposed to both human hunting and large carnivore predation, which often have opposing spatiotemporal activity peaks. In multi-predator landscapes, the ability of prey to avoid both hunters and large carnivores spatially may decrease (Lone et al. 2014). However, contrasting temporal activity of hunters and large carnivores may provide prey with the opportunity to adjust their behaviour in response to seasonal and diurnal risk patterns (Monterroso et al. 2014; Lone et al. 2017). Improving our understanding of the risk effects from both humans and large carnivores is crucial for managing ungulate populations, as behaviours aimed at minimizing risk exposure may also affect demographic traits like growth and reproduction.

## Supplementary Information

Below is the link to the electronic supplementary material.

Supplementary file1 (DOCX 498 KB)

## Data Availability

All data and code used in this study are publicly available in the DataVerse repository at: 10.18710/OAT6O9.
