# Supplementary material for: Contrasting risk patterns from human hunters and a large carnivore influence the habitat selection of shared prey
Source: Oecologia. 2025 Jul 1;207(7):118. doi: 10.1007/s00442-025-05742-z (PMC12213882; doi:10.1007/s00442-025-05742-z)

Contrasting risk patterns from humans and a large carnivore influence the habitat selection of shared prey

# **Supplementary material**

Table 1: Generalized Additive Models investigating non-linear patterns of habitat use of moose from September to April in south-central Scandinavia. Relative hunting risk and wolf predation risk of used GPS locations from moose were modelled as a function of time (number of days from the 15^th^ of August), time of day (day/night) and the two-way interaction. Each response variable (hunting and wolf predation risk) was modelled separately. Smoothed terms are represented by an *s* before the variable’s name. Effective degrees of freedom (edf), F-value and p-values are provided for each model. The variables are s(diff), which represents the number of days since the 15^th^ of August, daylight (day or night) and s(year, id), which represents the random effects of year and moose ID.

## **Table 1**

| **Hunting risk** | | | |
| --- | --- | --- | --- |
| Variable | Edf | F-value | p-value |
| s(diff) | **10.48** | 1307.8 | <0.001 |
| s(diff):daylightNight | **0.79** | 340.2 | <0.001 |
| s(diff):daylightDay | **1.17** | 192.4 | <0.001 |
| s(year, id) | **22.98** | 1238 | <0.001 |
| **After the hunting period** | | | |
| Variable | β estimate | Standard error | p-value |
| s(diff) | **8.39** | 706 | <0.001 |
| s(diff):daylightNight | **2.00** | 394 | <0.001 |
| s(diff):daylightDay | **0.52** | 1706 | <0.001 |
| s(year, id) | **22.93** | 408 | <0.001 |

Figure 1: *Examples of different moose home ranges during and after the hunting season in south-central Scandinavia during fall-winter 2019/20.* The moose population within the study area is partially migratory, meaning that some moose migrate between summer and winter home ranges while others are stationary throughout the year. Shaded polygons represent home ranges during the main moose hunting season, whereas empty polygons correspond to moose home ranges after the hunting season (and different colours represent different individuals). In the figure, we present the three common scenarios within our study area: 1) stationary moose have mostly overlapping home ranges (green and yellow) with different degrees of overlap (ranging from 50% to 100% overlap); 2) migratory moose have a summer-autumn home range that corresponds with the hunting season (pink and red) and the winter home ranges match with the after hunting season home range (0% overlap); and 3) migratory moose have a summer-autumn home range that matches the hunting season but these moose migrate before the end of the hunting season (blue individual), which means that they have two *hunting season* home ranges (two polygons) and one *after the hunting season* home range. We did not include migration

positions in the analysis. During and after the hunting season, the average home range size was 35.5 km^2^ and 23.5 km^2^, respectively.

# **Figure 1**


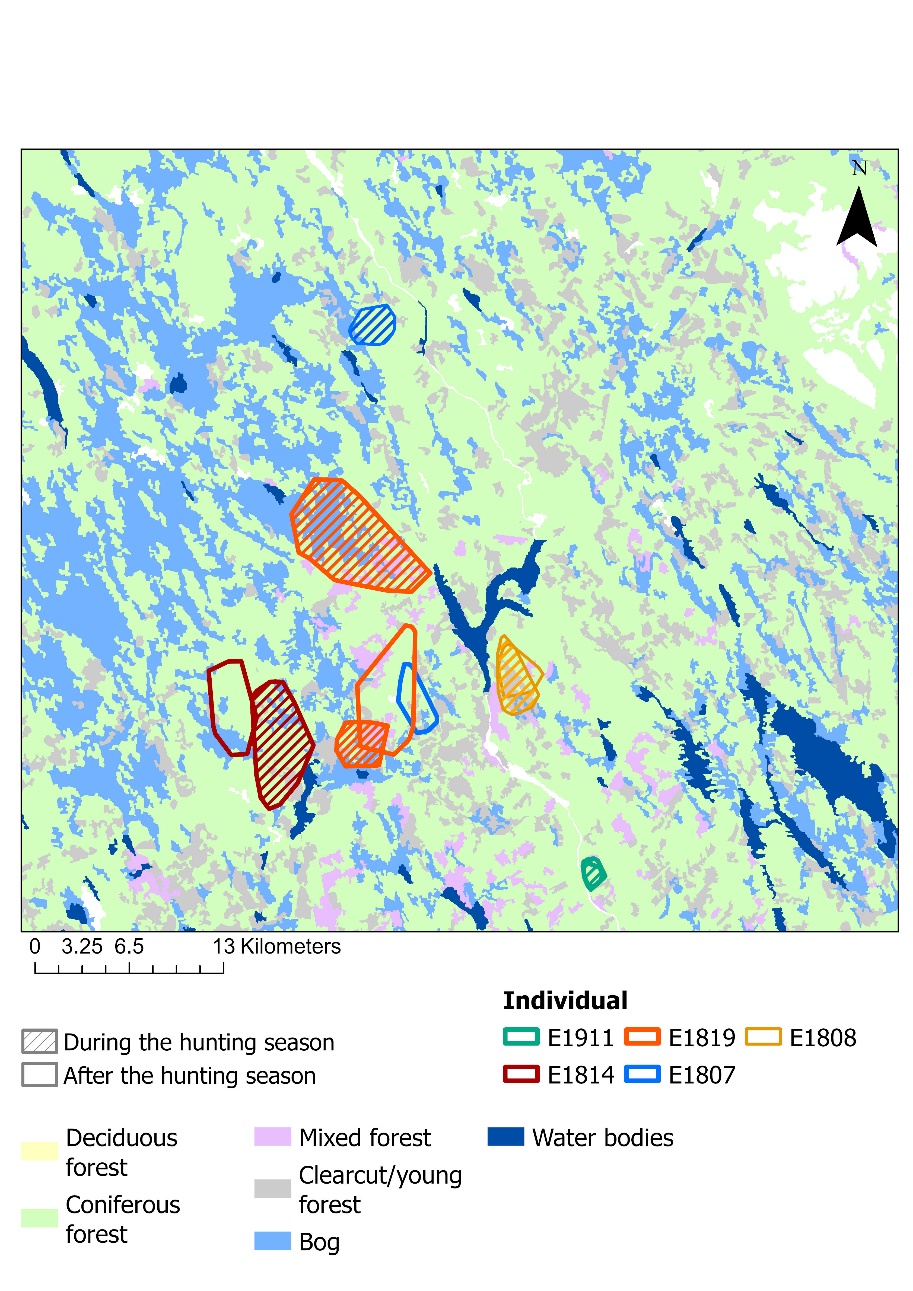

Supplement: Supplementary file 1 — Supplementary file1 (DOCX 498 KB) [file 442_2025_5742_MOESM1_ESM.docx]
